# Effects of Low-Dye Tape on Arch Height and Its Impact on the Medial Gastrocnemius Electromyographic Activity in Structurally Differentiable Foot Types: A Cross-Sectional Observational Study

**DOI:** 10.3390/life13122309

**Published:** 2023-12-08

**Authors:** Carlos Martínez-Sebastián, Laura Ramos-Petersen, María Gámez-Guijarro, Raquel Alabau-Dasi, George Banwell, Almudena Núñez-Fernández, Rubén Sánchez-Gómez, Álvaro Gómez-Carrión

**Affiliations:** 1 Nursing and Podiatry, University of Malaga Facultad de Ciencias de la Salud, 29071 Malaga, Spain; carlos_mar_seb@hotmail.com (C.M.-S.); lrpetersen@ucam.edu (L.R.-P.); mgamez303@gmail.com (M.G.-G.); raquelalabau12@gmail.com (R.A.-D.); gbanwell@hotmail.com (G.B.); 2 Nursing Department, Faculty of Nursing, Physiotherapy and Podiatry, University Complutense of Madrid, 28040 Madrid, Spain; almnun01@ucm.es (A.N.-F.); alvaroalcore@hotmail.com (Á.G.-C.); 3 Fundación para la Investigación Biomédica del Hospital Clínico San Carlos (FIBHCSC), Health Research Institute San Carlos Clinical Hospital (IdISSC), 28040 Madrid, Spain

**Keywords:** foot, tape, arch height, EMG, medial gastrocnemius, low-Dye tape

## Abstract

Background: Low-Dye tape (LDT) is a short-term treatment for plantar fasciitis, where external stabilization by means of the tape improves kinetics, kinematics, pain level, and electromyography (EMG). Purpose: The purpose of this study was to compare the EMG of the medial gastrocnemius (MG) and changes in arch height (AH) based on the type of foot. Methods: A total of 30 subjects participated in this study; they walked on a treadmill barefoot and when taped, where the average activity and changes in AH were measured over a 30 s period. The statistical intraclass correlation coefficient (ICC) to test for reliability was calculated, and the Wilcoxon test was determined for measures of EMG and AH. Results: The reliability of the values of EMG was almost perfect. The data show that there was an increase in height in the comparison of the moment pre-baseline walking and post-taped walking on neutral feet (5.61 ± 0.46 vs. 5.77 ± 0.39 cm, *p* < 0.05), on pronated feet (5.67 ± 0.57 vs. 6.01 ± 0.53 cm, *p* < 0.001) and on supinated feet (5.97 ± 0.36 vs. 6.28 ± 0.27 cm, *p* < 0.05). In the MG, EMG activity decreased significantly in the taped condition compared to the baseline condition in neutral subjects (0.0081 ± 0.016 vs. 0.076 ± 0.016 mV, *p* < 0.05) and in pronated subjects (0.081 ± 0.022 vs. 0.068 ± 0.025 mV, *p* < 0.05). Conclusions: It was demonstrated that with the use of LDT, there was an improvement in the average activity in the MG in pronated and neutral feet. All foot types improved in arch height with the use of tape.

## 1. Introduction

The two gastrocnemii constitute the most superficial musculature of the posterior aspect of the leg. These muscles have a proximal origin in the femoral condyles, with the medial twin having more volume and more caudal extension than the lateral one. The myotendinous union of both creates a deep aponeurosis that joins the superficial aponeurosis of the soleus to form the Achilles tendon (AT); the myotendinous union is about 10–15 cm from its insertion in the posterior tubercle of the calcaneus [1]. These fibers form a continuation of the fascia. This is a fibrous structure of connective tissue that extends from the calcaneus to the proximal phalanx of the toes [2]. Studies have shown that there is an anatomical, histological, mechanical and functional link between the plantar fascia and the AT through the calcaneal tuberosity [3].

Some researchers have shown that excessive stretching of the AT, which is the result of intense muscle contraction, is a plausible mechanical factor for excessive weight bearing by the plantar fascia due to marked tensile strength [3]. This increase in tension can generate injury to the Achilles–calcaneus–plantar system [4]. The AT, when inserted medial to the axis of the subtalar joint, has a decelerating role in this joint [5].

It has been recognized that foot posture influences the risk of injuries in sports [6]. It has been suggested that in flattened arches, the need for muscular support is responsible for stability; once fatigued, it can lead to injuries such as stress fractures in the tibia [7]. Systematic reviews show evidence indicating that pronated foot posture is associated with greater electromyography (EMG) amplitude for the inverter muscles. There are several elements, such as foot type and the device used, that could introduce possible interferences in these measurements. A recent review of the literature highlighted the importance of carrying out research with more rigorous methodologies in this area [6]. Studies like that of Telfer et al. [8] have shown that foot type interferes in the muscular activity of the biceps femoris, vastus lateralis and vastus medialis through customized foot orthoses.

The use of “low-Dye tape” (LDT) is a technique that has been used by clinicians since 1941; it was first described by Dye [9] and later modified by various authors [10]. Tape that extends to the leg is known as “high-Dye tape” (HDT), while isolated tape to the foot without crossing the ankle is called “LDT”. LDT is a common conservative treatment, particularly in the short term, for fasciopathy. This tape reduces pain [11], improves plantar pressures [12], reduces pronation [13] and decreases the average muscle activity [14].

Previous studies have shown that tape generates a decrease in the fall of the internal longitudinal arch. There is controversy about whether the electromyographic activity of the medial gastrocnemius (MG) increases or decreases with HDT [15,16,17].

We hypothesize that tape can decrease the activity of the MG depending on foot type. The decelerating function of the Achilles and its close relationship with the fascia makes us consider whether pronated foot types would benefit from a reduction in the muscular activity of the GM through LDT. No previous studies have investigated the effects of LDT in structurally differentiable foot types, defined using the foot posture index (FPI). Therefore, the purpose of this investigation was to determine if the effect is different on EMG muscle activity with LDT, as well as its effects on arch height (AH) in different foot types (pronated, supinated and neutral).

## 2. Materials and Methods

The research team presented their project to the Vírgen Macarena-Virgen del Rocío University Hospital, which is qualified as a public institution, to request approval from the ethics committee (f7f4a6567676d7ba7163bce0d15e7f98c9f32356). The ethical criteria for human research were followed in accordance with the Declaration of Helsinki, and signed informed consent was obtained from all subjects.

### 2.1. Design and Sample Size

In this cross-sectional observational study, the statistics and research software EPIDAT 3.0 (https://www.sergas.es/Saude-publica/EPIDAT?idioma=es, accessed on 22 June 2021) from a public university was used to calculate the sample size and to detect any measurable differences in medial gastrocnemius muscle activity with and without LDT during gait. In a previous study on surface electromyography of the MG, 0.208 mV was the highest value for an 8 mm rocker arm orthosis, and 0.032 mV was the lowest value for an 8 mm Morton’s Typical Extension Orthosis for the medial gastrocnemius [18]. Taking into account a statistical power of 80%, β = 20%, a confidence interval (CI) of 95% and α = 0.05, 30 subjects were required to complete the study. The criteria for Strengthening the Report of Observational Studies in Epidemiology were followed, and a consecutive random sampling technique was employed [19].

### 2.2. Participants

Participants were recruited from a biomechanical clinic (Carlos’s clinic), in Murcia, Spain, for a period of 3 months (December 2020–February 2021). A total of 30 subjects (10 neutral feet (5 women and 5 men), 10 pronated feet (5 women and 5 men) and 10 supinated feet (5 women and 5 men)) were invited to participate in the study, and their eligibility was assessed. The dominant foot of all participants was chosen. The following inclusion criteria were used to select the participants: (1) healthy male and female participants, between 24 and 55 years old and without injury or pain at the time of the test; (2) individuals with an FPI between −4 and +9. They were then divided into the following groups: neutral subjects, with an FPI of 0 to 5; pronated subjects, with an FPI of 6 to 9; and supinated subjects, with an FPI of −1 to −4 [20]. The following exclusion criteria were used: previous surgery on the lower limbs, neurological conditions, history of heart problems, allergy to adhesive tape, previous experience with antipronation tape, and never having walked on a treadmill. All participants were informed about the procedures and provided signed consent. Body mass index (BMI) was considered to select a homogeneous sample, applying the Quetelet equation as BMI = weight (kg)/height (m)^2^ [21].

### 2.3. Instrumentation and Evaluations

**Figure 1:**
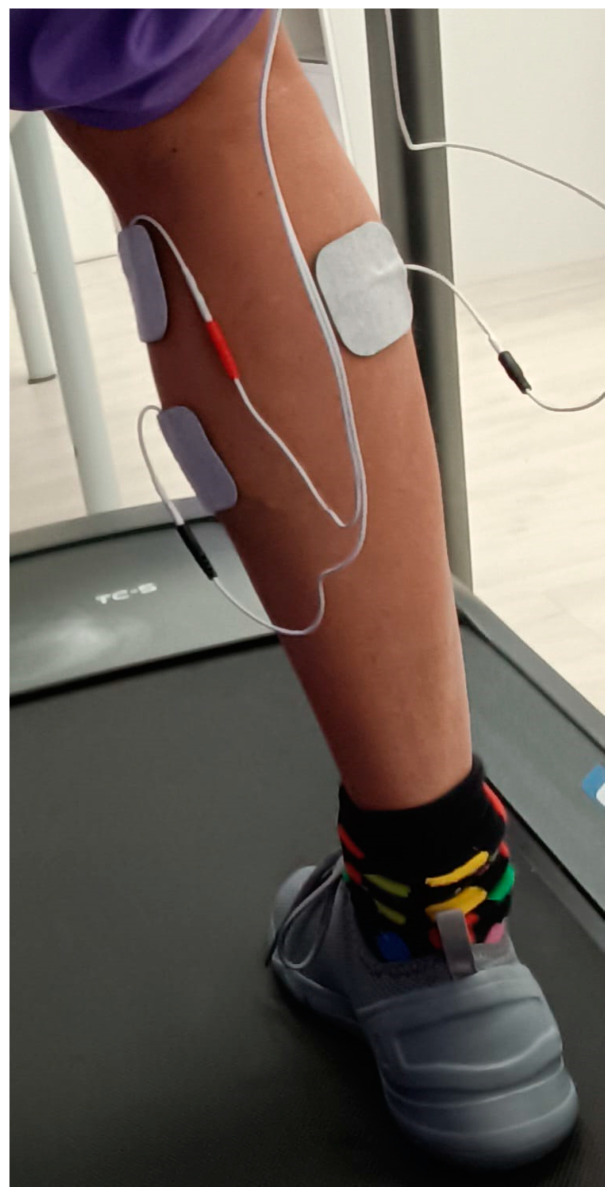
Locations of the electrodes for assessing the medial gastrocnemius.

To investigate the average activity of the MG muscle during a walking test, the researchers employed the Neurotrac^®^ Simplex Plus, an electronic EMG device, manufactured by Verity Medical Ltd. in Braishfield, UK. This device features USB–Bluetooth connectivity [22] for data transfer and analysis. The recording range of the Neurotrac^®^ Simplex Plus device spans from 0.0002 mV to 0.2 mV. It possesses a sensitivity of 0.0001 mV root mean square (RMS) and allows for a wireless connection range of 10 m through Bluetooth. The device maintains an accuracy of 4% of the mV reading, with an additional margin of ±0.0003 mV at a frequency of 200 Hz. It employs a bandpass filter that ranges from 18 Hz ± 4 Hz to 370 Hz ± 10% for readings below 235 mV. To capture the electrical action of the muscle fibers, the researchers employed circular surface electrodes with a diameter of 30 mm. These electrodes were self-adhesive and comprised high-quality hydrogel and a conductive carbon film. The receiver module of the Neurotrac^®^ device captured the signal from each electrode, which was subsequently processed and filtered automatically by the accompanying Neurotrac^®^ software 5. In this study, the mean of the walking gait cycle was measured as in previous studies of lower limbs (Figure 1) [18,23].

The FPI, validated by Redmond et al. in 2006 [20], is an observational tool that takes into account the three-dimensionality of the foot and its biomechanical complexity. The FPI has been shown to have good intrarater reliability (ICC = 0.928 to 0.937) and moderate interclinician reliability (ICC = 0.525 to 0.655) [18]. The FPI provides a total value from −12 points (highly supinated) to +12 points (highly pronated).

AH measurements were performed as an indication of foot posture and changes caused by the tape. This foot posture measurement was performed due to its validity and high reliability [24]. The participant stood on a piece of paper with weight evenly distributed between both feet; an outline was then drawn around both feet to create a draft. The draft ensured a standardized and repeatable foot position. A digital caliper (Mitutoyo^®^, Kawasaki, Japan) was used to measure AH as the vertical distance from the insole (ground) to the dorsal surface of the midfoot (i.e., 50% of the foot length).

### 2.4. Materials

**Figure 2:**
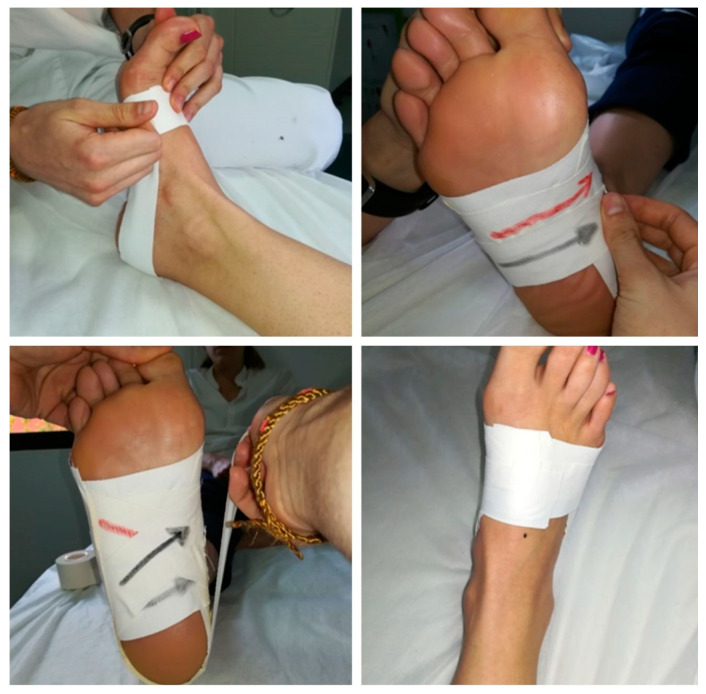
Modified low-Dye tape.

The taping method used was the modified low-Dye tape method described by Schulties and Draper [25]. The taping procedure was as follows: Each subject was placed in a supine position with the foot free to apply the tape. An initial anchor was placed around the metatarsal heads. Next, the 2nd anchorage was initiated on the lateral aspect of the head of the 5th metatarsal, surrounding the foot up to the head of the first metatarsal, performing a dorsiflexion of the 1st metatarsophalangeal joint to plantarflex the first metatarsal. From the lateral aspect, strips were added to the medial area to provide stability. The anchors were repeated again. This was followed by the addition of strips around the forefoot to give more rigidity to the tape. The taping technique was completed by applying strips around the transverse plane of the foot, from the dorsomedial aspect of the first metatarsal head to the dorsolateral aspect of the fifth metatarsal head (Figure 2).

### 2.5. Procedure

The assessment of the foot posture was carried out by measuring the FPI with the subjects barefoot, in a standing position. The clinician then performed a physical evaluation of the subjects and applied the eligibility criteria. To visualize the muscle belly of the MG, each subject was asked to plantar flex their foot. The surface electrodes were then placed longitudinally over the most prominent region of the MG, according to the “European recommendations for surface EMG” [26]. Subjects were then asked to stand on one leg in a tip-toe position, using their previously chosen foot for 5 seconds to set the maximum voluntary contractions to calibrate the software and normalize the amplitudes of the EMG data for each test [27]. The mean EMG peroneus muscle activity pattern of the dominant foot was recorded 3 times for 30 seconds each, leaving 5 minutes of rest between each test.

**Figure 3:**
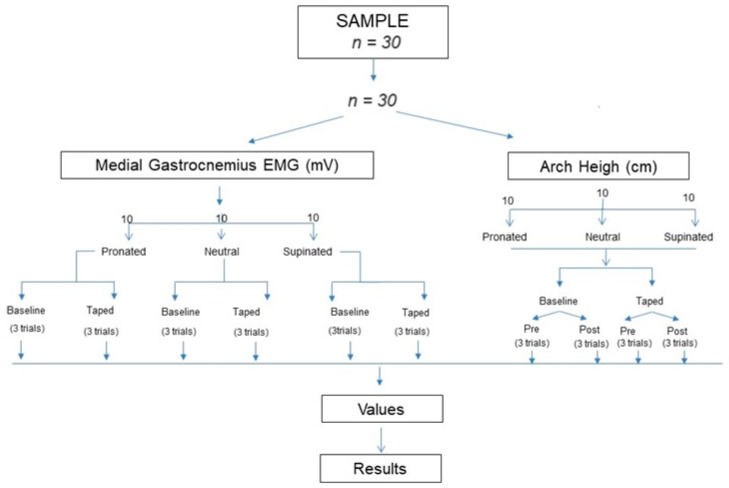
Flow chart. Abbreviations: electromyography (EMG), millivolts (mV), centimeter (cm).

For baseline measurements, participants were then asked to walk on a treadmill (BH, I.F2W DUAL G6473UW) in “New Feel PW 100 M medium grey” shoes (reference number: 2018022) for an acclimatization period of 5 minutes. The order of exposure to the condition was not randomized, because the non-treadmill (baseline) trials performed after the treadmill trials were deemed not to be representative of the actual walking pattern of the participants, due to a possible dragging effect caused by the treadmill [28]. Participants started at a self-selected speed, without an incline, and then the speed was gradually increased until reaching the test speed of 4.5 km/h [18]. At this point, each participant walked for 10 min while EMG data were recorded. The treadmill and the EMG were then stopped, the participants took off their shoes and the LDT technique was applied to the dominant foot. Then, participants put on their shoes, and the test was resumed for another 10 minutes at the same speed of 4.5 km/h while EMG data were recorded. AH measurement was performed 3 times before and after the 10-minute walk without the LDT and immediately after the 10-minute walk with the LDT (Figure 3).

### 2.6. Statistical Analysis

To test the reliability of the present investigation, the intraclass correlation coefficient (ICC) and the standard error of measurement (SEM) were calculated for the subjects in the 2 conditions for the MG during the walking test [18]. According to Landis and Koch [24], ICC coefficients less than 0.20 indicated slight agreement, 0.20–0.40 indicated fair reliability, 0.41–0.60 indicated moderate reliability, 0.61–0.80 indicated substantial reliability, and 0.81–1.00 indicated near-perfect reliability. The authors considered appropriate coefficients ≥0.81 to determine that the results were valid. The SEM was used to evaluate the minimum detectable change (MDC) for all measurements. This is known as the Reliable Index of Change (RCI) and was used to determine the clinical significance of the data. The Shapiro–Wilk test was used to assess the normality of the sample, and a normal distribution was presented if *p* > 0.05. Demographic values were presented as mean and standard deviation (±SD). Continuous variables (i.e., age, height, weight and BMI) were compared using the independent t-test, and categorical demographic variables (i.e., gender) were compared using the chi-square test. The *p*-values of the multiple comparisons were corrected with a paired nonparametric Friedman test to show that all the taped and baseline conditions were different from each other. The Wilcoxon test with Bonferroni correction was performed to analyze the differences between the five different conditions, indicating statistically significant differences when *p* < 0.05 with a 95% CI. All the values that were generated using NeuroTrac^®^ software 5 were loaded into an Excel^®^ template (Windows^®^ 97–2003) and analyzed with the SPSS program version 19.0 (SPSS Science, Chicago, IL, USA).

## 3. Results

The Shapiro–Wilk test showed a non-normal distribution of the sample (*p* < 0.05), while the Friedman test showed that the values were different between the four conditions in AH and two conditions in EMG (*p* < 0.05).

Thirty-six subjects were asked to participate in the experiment, and their eligibility was assessed; six did not meet the study entry requirements. Ultimately, 30 participants (15 men and 15 women) were enrolled in the study. Sociodemographic data are shown in Table 1. The mean age, BMI, height, weight and gender ratios of the three groups did not differ (*p* > 0.27).

The reliability of the data obtained from the EMG activity of the muscles in the taped and baseline conditions and the arch height during gait in the four different conditions were presented as ICC and SEM values, which are shown in Table 2 and Table 3. Most of the values reached cut-off values greater than 0.98 in the ICC data in EMG, and 0.997 in arch height, suggesting “near-perfect reliability”, with 0.994 for EMG taped as the highest value and 0.980 for EMG baseline as the lowest value for the MG, and 0.998 for arch height both after baseline and taped walking as the highest value and 0.997 for arch height both before baseline and taped walking as the lowest value. For the SEM, 0.020 cm was the lowest value established for arch height after taped walking, 0.027 cm was the highest value for arch height for pre-taped walking, 0.001 mV was the lowest value for taped walking and 0.003 mV was the highest value of baseline for muscle activity of the MG. The highest value of MDC for arch height was 0.075 cm before baseline walking, and 0.054 cm was the lowest value after taped walking. The highest value of MDC was 0.008 mV baseline walking, and 0.001 mV was the lowest value that occurred during taped walking for the MG activity.

### 3.1. Arch Height

The AH and the comparison values of the measured conditions before and after baseline and taped walking are shown in Table 4. There was a significant decrease in height in comparison to the moment before and after baseline walking on neutral feet (5.61 ± 0.46 vs. 5.4 ± 0.43 cm, *p* < 0.001), pronated feet (5.67 ± 0.57 vs. 5.43 ± 0.58 cm, *p* < 0.001) and supinated feet (5.97 ± 0.36 vs. 5.87 ± 0.31 cm, *p* < 0.05). There was a significant increase in height in baseline and taped feet before walking on neutral feet (5.61 ± 0.46 vs. 5.92 ± 0.46 cm, *p* < 0.05), pronated feet (5.67 ± 0.57 vs. 6.12 ± 0.53 cm, *p* < 0.001) and supinated feet (5.97 ± 0.36 vs. 6.43 ± 0.32 cm, *p* < 0.05). There was an increase in height in comparison to the moment before baseline walking and after taped walking on neutral feet (5.61 ± 0.46 vs. 5.77 ± 0.39 cm, *p* < 0.05), pronated feet (5.67 ± 0.57 vs. 6.01 ± 0.53 cm, *p* < 0.001) and supinated feet (5.97 ± 0.36 vs. 6.28 ± 0.27 cm, *p* < 0.05). There was a significant decrease in height in the comparison of the moment before and after taped walking on neutral feet (5.92 ± 0.46 vs. 5.77 ± 0.39 cm, *p* < 0.001), pronated feet (6.12 ± 0.53 vs. 6.01 ± 0.53 cm, *p* < 0.001) and supinated feet (6.43 ± 0.32 vs. 6.28 ± 0.27 cm, *p* < 0.001).

### 3.2. EMG

The mean muscle EMG activity in the MG compared between taped and baseline conditions is presented in Table 5. In the MG, EMG activity decreased significantly in the taped condition compared to the baseline condition on neutral feet (0.081 ± 0.016 vs. 0.076 ± 0.016 mV, *p* < 0.05) and on pronated feet (0.081 ± 0.022 vs. 0.068 ± 0.025 mV, *p* < 0.05); on the other hand, there was no statistical relevance in the EMG activity of the MG among the supinated feet (*p* = 0.640).

## 4. Discussion

This study aimed to evaluate the effect of LDT on AH and the average muscle activity in different structurally differentiable foot types to discover the changes in AH and EMG of the MG during walking.

### 4.1. Arch Height

Due to the wide variability of AH nomenclature found in the literature (navicular drop, height of the navicular, height of the internal longitudinal arch), it was difficult to establish a concrete comparison between our obtained results and those of other authors [12,14,17].

**Figure 4:**
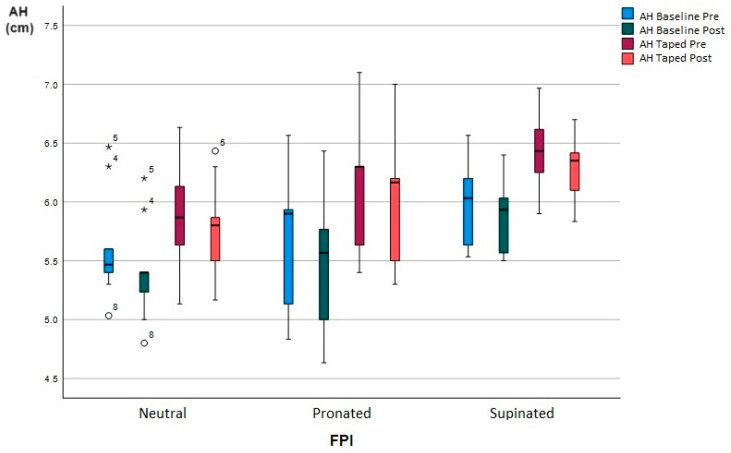
Effect of LDT on arch height (AH). Whisker diagram of arch height (AH). Average of the 4 conditions, AH baseline before walking, AH baseline after walking, AH taped before walking, AH taped after walking. Asterisk, high outlier. Circle, low outlier.

In the present study (Figure 4), foot type was taken into account, and it was found that there was a decrease in the arch height after walking 10 minutes without taping, on neutral feet, of 0.21 cm (3.7%) (*p* < 0.001); on pronated feet, of 0.24 cm (4.2%) (*p* < 0.001); and on supinated feet, of 0.1 cm (1.6%) (*p* < 0.05). These data are in line with the study carried out by Cornwall, Mark W. and McPoil, Thomas G. on the relationship between static foot posture and foot mobility, which found that individuals with higher FPI values demonstrated greater mobility than those with lower FPI values [29].

In the 2021 meta-analysis performed by Meihua Tang and Lin Wang et al. [30], all types of tape were found to significantly increase the height of the navicular immediately after taping compared to the baseline (*p* < 0.001); in the review by Melinda Franettovich et al. [10], an 8 to 16% increase in navicular height was found. In our study, we found that there was an increase in the arch height with tape, compared to the reference values on neutral feet of 5.5% (*p* < 0.05); pronated, 7.9% (*p* < 0.001); and supinated, 7.7% (*p* < 0.05). Contrary to this, we found an article where taping did not result in a better arch height immediately after application, although this could be due to the use of a more elastic tape [31]. Another cause that may make it less effective could be the loss of the traction quality of the tape or the loss of adherence due to movement or sweat.

In the study by Clayton F. Holmes et al., the LDT maintained the initial effects after walking for 10 min when recently applied (16.7% to 10.2%), but its effectiveness was lost during jogging [32]. The study by Melinda Franettovich [10], who used augmented Dye tape, had a mean increase in AH of 8 mm (12.9%) immediately after the application of the tape (*p* = 0.005); although the effectiveness was reduced by 5mm (8.5%) after 10 minutes of walking, it was still higher than the reference values (*p* = 0.002). 

In our study, subjects with neutral feet that were taped presented an efficacy of 2.8% (*p* = 0.005), which decreased by 2.7% compared to the start of the taping; pronated feet presented an efficacy of 5.9% (*p* < 0.001), which decreased by 2% compared to the start of the taping; and supinated feet presented an efficacy of 5.1% (*p* = 0.005), which decreased by 2.6% compared to the start of the taping. In all the conditions, the efficacy increased compared to the reference values.

### 4.2. Electromyography

**Figure 5:**
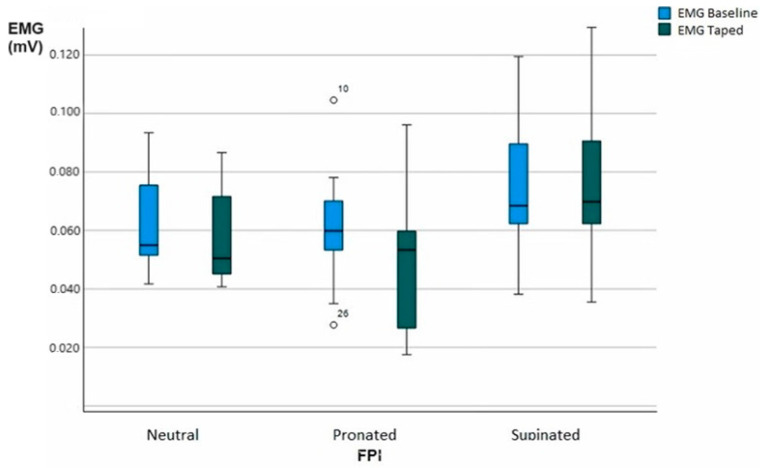
Effect of LDT on electromyography (EMG) (mV). Whisker diagram of electromyography (EMG). Average of 2 conditions, EMG baseline and EMG taped mean. Circle, low outlier.

There were no studies that evaluated the LDT and EMG of the MG; on the other hand, we found studies that used antipronation tape to analyze this muscle. A study by Franettovich et al. [16] confirmed these effects, observing a decrease in MG muscle activity during the stance phase of gait, over a period of 10 min, in subjects with pain. On the other hand, another study by the same author did not obtain any change in the activity of the MG after the application of the tape. In studies where plantar supports with medial wedges were used in the hindfoot, there was an improvement in the activity of the MG due to the passive stability provided by the template, reducing the demand on the MG muscle [33]. In our study (Figure 5), we found that with the stability provided by the tape, we obtained a significant difference of 5.9% (*p* < 0.05) in the average MG activity on neutral feet and 15% (*p* < 0.05) in the average MG activity on pronated feet. Although there were no statistically significant results for supinated feet, it is interesting to highlight the fact that supinated feet did not obtain any improvement in the mean MG activity.

### 4.3. Clinical Implication

Adrienne E Hunt et al. [34] confirmed that there was a greater activity of the MG in the middle phase of gait in subjects with pronated feet with respect to neutral feet, causing a greater muscular effort to stabilize the arch. This tape could reduce arch drop, generating passive stability in all types of feet; in all of them, there were improvements compared to the reference values. LDT was originally intended to reduce pain in patients with plantar fasciitis. The results from previous studies inform us that a decrease in the drop of the arch of 0.1 to 0.3 cm could reduce this pain from 23 to 91% [35]. Another study confirmed a close relationship between MG stiffness and pain in patients with plantar fasciitis, and it is recommended to reduce the stiffness of the MG in the treatment of patients with plantar fasciitis [36]. Regarding EMG, we only found significant differences in pronated and neutral feet; we found that these types of feet have a better mean MG activity. With these results, this tape is not only important in the treatment of plantar fasciopathy, but it may also play a fundamental role in the short-term treatment of lesions of the entire Achilles–calcaneal–plantar system, due to the reduction in the MG’s activity.

### 4.4. Limitations

This study has a few limitations. It would have been interesting to be able to measure the lateral gastrocnemius at the same time. The present results are not specific to any phase of walking gait, but to a mean value of a whole walking gait cycle. The results must be taken with caution since they correspond to the dynamic global muscular activity of walking and not to a specific muscular activity of any phase. There is no recent literature on the change in muscle activity with the use of taping. Finally, step length was not taken into account in case it could differ from one condition to another.

### 4.5. Future Lines of Investigation

In the future, the same research could be carried out on subjects with fasciopathy, and the results could be compared with healthy subjects. Other muscles such as the tibialis posterior and the lateral gastrocnemius could also be included. Finally, it would be informative to evaluate the phases of walking separately.

## 5. Conclusions

This study is the first to evaluate the effect of LDT on AH and the average EMG activity of the MG, classified with FPI scores (supinated, pronated and neutral). We found evidence that LDT reduces average MG EMG in subjects with pronated and neutral feet when walking. It was confirmed together with previous findings that taping not only increases AH in all subjects at rest relative to barefoot without walking, but also does not lose effectiveness after 10 minutes of walking if we compare it with the reference values.

## Figures and Tables

**Table 1:** Anthropometric participant characteristics (sample size = 30 participants).

Variable	*n* = 30 Mean ± SD (95% CI)
Age (years)	33.85 ± 10.25 (24–55)
Weight (kg)	73.08 ± 13.89 (54–103)
Height (m)	1.74 ± 0.08 (1.58–1.92)
BMI (kg/m^2^)	23.86 ± 3.10 (19.60–31.14)

Abbreviations: SD = standard deviation; CI = confidence interval; BMI = body mass index.

**Table 2:** ICC reliability of arch height variables before and after baseline and taped walking.

Variable	Baseline Pre (cm)			Baseline Post (cm)			Taped Pre (cm)			Taped Post (cm)		
	ICC (95%CI)	SEM	MDC	ICC (95%CI)	SEM	MDC	ICC (95%CI)	SEM	MDC	ICC (95%CI)	SEM	MDC
AH	0.997 (0.995–0.999)	0.025	0.070	0.998 (0.996–0.999)	0.022	0.060	0.997 (0.994–0.999)	0.027	0.075	0.998 (0.996–0.999)	0.020	0.054

Abbreviations: AH = arch height; ICC = intraclass correlation coefficient; CI = confidence interval; SEM = standard error of measurement; MDC = minimal detectable change; baseline pre = assessments before walking without low-Dye tape; baseline post = assessments after walking without low-Dye tape; taped pre = assessments before walking with low-Dye tape; taped post = assessments after walking with low-Dye tape.

**Table 3:** ICC reliability of baseline and taped electromyography variables.

Variable	Baseline			Taped		
	ICC (95%CI)	SEM	MDC	ICC (95%CI)	SEM	MDC
EMG MG (mV)	0.980 (0.962–0.990)	0.003	0.008	0.994 (0.989–0.997)	0.001	0.005

Abbreviations: EMG = electromyography; MG = medial gastrocnemius; ICC = intraclass correlation coefficient; CI = confidence interval; SEM = standard error of measurement; MDC = minimal detectable change; mV = millivolts; baseline = without low-Dye tape; taped = with low-Dye tape.

**Table 4:** Arch height (AH) and comparison values of the measurement conditions before and after baseline and taped walking.

Variable AH *n* = 30	Baseline Pre Mean (cm) ± SD (95% CI)	Baseline Post Mean (mV) ± SD (95% CI)	Taped Pre Mean (mV) ± SD (95% CI)	Taped Post Mean (mV) ± SD (95% CI)	*p*-Value Baseline Pre vs. Baseline Post	*p*-Value Baseline Pre vs. Taped Pre	*p*-Value Baseline Pre vs. Taped Post	p-Value Taped Pre vs. Taped Post
Neutral	5.61 ± 0.46	5.4 ± 0.43	5.92 ± 0.46	5.77 ± 0.39	<0.001 **	<0.05 *	<0.05 *	<0.05 *
	(5.03–6.46)	(4.80–6.20)	(5.13–6.63)	(5.17–6.43)				
Pronated	5.67 ± 0.57	5.43 ± 0.58	6.12 ± 0.53	6.01 ± 0.53	<0.001 **	<0.001 **	<0.001 **	<0.001 **
	(4.83–6.57)	(4.63–6.43)	(5.40–7.10)	(5.3–7.0)				
Supinated	5.97 ± 0.36	5.87 ± 0.31	6.43 ± 0.32	6.28 ± 0.27	<0.05 *	<0.05 *	<0.05 *	<0.001 **
	(5.53–6.57)	(5.50–6.40)	(5.90–6.97)	(5.83–6.70)				

Abbreviations: cm = centimeters; ± SD = standard deviation; *p* < 0.05 * (95% CI) was considered statistically significant; *p* < 0.001 ** (95% CI) was considered statistically significant. Baseline pre = assessments before walking without low-Dye tape; baseline post = assessments after walking without low-Dye tape; taped pre = assessments before walking with low-Dye tape; taped post = assessments after walking with low-Dye tape.

**Table 5:** Signal amplitudes and comparison values of the mean EMG activities of the MG muscles in baseline and taped conditions.

Variable EMG *n* = 30	Baseline Mean (mV) ± SD (95% CI)	Taped Mean (mV) ± SD (95% CI)	*p*-Value EMG Baseline vs. EMG Taped
Neutral	0.081 ± 0.016	0.076 ± 0.016	<0.05 *
	(0.061–0.113)	(0.060–0.106)	
Pronated	0.081 ± 0.022	0.068 ± 0.025	<0.05 *
	(0.047–0.124)	(0.037–0.116)	
Supinated	0.094 ± 0.025	0.096 ± 0.027	0.640
	(0.058–0.139)	(0.055–0.149)	

Abbreviations: MG = medial gastrocnemius; EMG = electromyography; mV = millivolts; ± SD = standard deviation; *p* < 0.05 * (95% CI) was considered statistically significant. Baseline = without low-Dye tape; taped = with low-Dye tape.

## Data Availability

The datasets used and analyzed during the current study are available from the corresponding author upon reasonable request.
